# Antidiabetic Drugs Can Reduce the Harmful Impact of Chronic Smoking on Post-Traumatic Brain Injuries

**DOI:** 10.3390/ijms24076219

**Published:** 2023-03-25

**Authors:** Farzane Sivandzade, Faleh Alqahtani, Hemangini Dhaibar, Diana Cruz-Topete, Luca Cucullo

**Affiliations:** 1 Department of Biological Sciences, Oakland University, Rochester, MI 48309, USA; 2 Department of Foundation Medical Studies, Oakland University William Beaumont School of Medicine, Rochester, MI 48309, USA; 3 Department of Pharmacology and Toxicology, College of Pharmacy, King Saud University, Riyadh 11362, Saudi Arabia; 4 Department of Molecular and Cellular Physiology, Louisiana State University Health Shreveport, Shreveport, LA 71103, USA

**Keywords:** oxidative stress, blood–brain barrier, traumatic brain injury, metformin, rosiglitazone, smoking

## Abstract

Traumatic Brain Injury (TBI) is a primary cause of cerebrovascular and neurological disorders worldwide. The current scientific researchers believe that premorbid conditions such as tobacco smoking (TS) can exacerbate post-TBI brain injury and negatively affect recovery. This is related to vascular endothelial dysfunction resulting from the exposure to TS-released reactive oxygen species (ROS), nicotine, and oxidative stress (OS) stimuli impacting the blood–brain barrier (BBB) endothelium. Interestingly, these pathogenic modulators of BBB impairment are similar to those associated with hyperglycemia. Antidiabetic drugs such as metformin (MF) and rosiglitazone (RSG) were shown to prevent/reduce BBB damage promoted by chronic TS exposure. Thus, using in vivo approaches, we evaluated the effectiveness of post-TBI treatment with MF or RSG to reduce the TS-enhancement of BBB damage and brain injury after TBI. For this purpose, we employed an in vivo weight-drop TBI model using male C57BL/6J mice chronically exposed to TS with and without post-traumatic treatment with MF or RSG. Our results revealed that these antidiabetic drugs counteracted TS-promoted downregulation of nuclear factor erythroid 2-related factor 2 (NRF2) expression and concomitantly dampened TS-enhanced OS, inflammation, and loss of BBB integrity following TBI. In conclusion, our findings suggest that MF and RSG could reduce the harmful impact of chronic smoking on post-traumatic brain injuries.

## 1. Introduction

Traumatic brain injury (TBI) is a broad term encompassing a vast array of mechanical injuries resulting in physical damage to the brain and is one of the most common causes of death and disability worldwide [1,2,3,4].

TBI is commonly categorized into primary and secondary injuries. Primary injury is the direct result of the physical damage promoted by the external mechanical forces to the brain tissue, impacting neurons, astrocytes, and blood vessels [5,6,7,8,9,10,11,12] and inducing a cascade of cellular and biochemical changes (secondary brain injury), leading to the onset of long-term post-traumatic neurological dysfunction [13,14]. The onset of secondary brain injury can start within days or years after the initial primary brain trauma and is coordinated by many pathophysiological mechanisms, including oxidative stress (OS), neuroinflammation, glutamate excitotoxicity, edema formation, mitochondrial dysfunction, etc. [15,16,17,18,19]. Among these, OS and neuroinflammation have been identified as key pathogenic factors prodromal to the onset of secondary brain injury [20,21], where pro-inflammatory mediators and the activation of innate immune responses in the central nervous system (CNS) [21,22,23,24,25,26] degrade the integrity of the blood–brain barrier (BBB; one of the remarkable risk factors directing the progression of injury and impacting the time course and the extent of neuronal repair) and may facilitate the invasion of more peripheral immune cells [27,28,29]. Furthermore, OS associated with inflammation exacerbates other pathological mechanisms at the cellular level, including the generation of lipid peroxidation, nuclear and mitochondrial DNA damage, neuronal apoptosis, etc. [2,30]. In this respect, tobacco smoking (TS) is a well-known premorbid risk factor that can exacerbate TBI severity and impair post-TBI recovery. The likely mechanism seems to correlate with vascular endothelial dysfunction in a causative and dose-dependent manner primarily linked to the TS content of ROS, nicotine, and OS-driven neuroinflammation [31,32], impacting the BBB and therefore promoting and/or aggravating the pathogenesis of several brain disorders [33] including TBI. Recently published in vitro and in vivo findings from our group strongly suggested that BBB impairment and increased severity of TBI following chronic TS exposure likely develop in response to common modulators such as OS, neuroinflammation, and alterations of the endogenous antioxidative response system (ARE) regulated by the nuclear factor erythroid 2-related factor (NRF2) [34]. Specifically, we determined that prolonged TS exposure negatively impacts the antioxidative response system (Nrf2 levels and activity), which also impacts the endothelial inflammatory responses, BBB integrity, and the plasma levels of thrombomodulin, a critical anticoagulant factor [35].

In recent years, the regulation of post-traumatic responses leading to elevated antioxidative and alleviated inflammatory responses has been reported to promote neuroprotective effects in TBI models [21,36,37,38,39,40]. Despite recent advances in TBI research, clinical trials have been less promising, and huge challenges to developing effective treatments remain unresolved [13]. We have recently shown that chronic TS and hyperglycemia carry similar risks for cerebrovascular diseases through sharing the same pathogenic mechanisms that involve common modulators of BBB impairment [41]. Furthermore, we demonstrated that antidiabetic drugs such as metformin (MF) and rosiglitazone (RSG) prevent/reduce BBB impairment caused by chronic TS exposure. MF is a first-line oral antidiabetic drug belonging to the biguanide class, which inhibits gluconeogenesis and thus suppresses hepatic glucose production. RSG, the thiazolidinedione compound, is well known to enhance insulin resistance through modulating adiponectin gene expression and is used for treating type-2 diabetes mellitus. RSG is also considered a transcription factor peroxisome proliferator-activated receptor (PPARγ) agonist [42]. Although the exact mechanism of MF and RSG is not fully understood, numerous studies have revealed that MF and RSG can potentially ameliorate oxidative damage [43,44,45,46]. For example, Tao et al. investigated the potential neuroprotective effects of MF on acute brain injury after TBI and assessed the underlying mechanisms [47]. Our data suggested that MF and RSG can effectively support counteractive antioxidative response mechanisms, drastically reducing TS-induced OS toxicity by activating the NRF2-ARE signaling pathway [48]. Thus, the objective of the present work was to evaluate the effectiveness of MF and RSG to prevent/reduce post-TBI exacerbation in response to chronic TS exposure and determine the key biological targets involved in the process. Our results suggest that the dysregulation of the cellular antioxidant response system caused by chronic exposure to TS is likely a major underlying mechanism worsening the severity of TBI and delaying post-TBI recovery. Our data also suggests that post-TBI treatment with MF or RSG could reduce the negative impact of chronic smoking on brain injury.

## 2. Results

### 2.1. Effect of MF and RSG on Body Weight and Fasting Blood Glucose Levels of Premorbid TS-Exposed and TBI-Induced Mice

**Figure 1:**
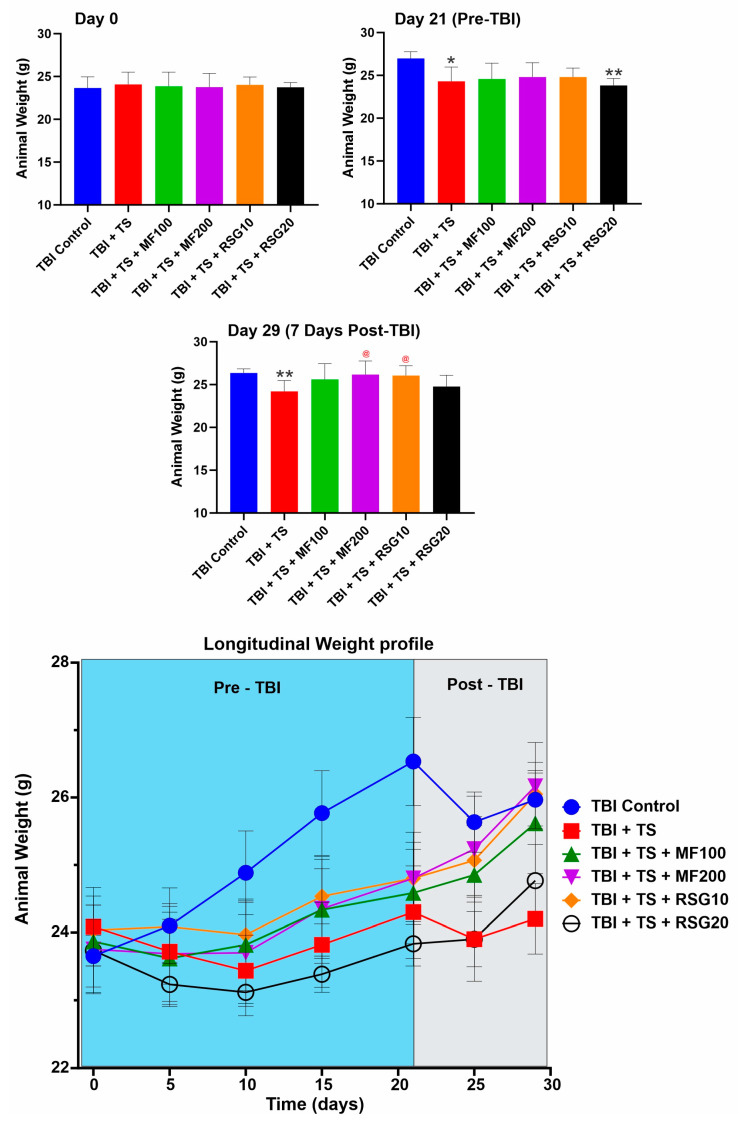
Effect of MF and RSG treatments on body weight of TS-exposed and TBI-induced mice. Measurements of animals’ body weight did not significantly differ between the tested groups at Day 0. However, at the end of the three weeks of exposure before TBI, animals exposed to TS showed decreased body weight compared to the control. Post-TBI body weight recovered following MF and RSG treatments for TBI. *n* = 6 biological replicates, * *p* < 0.05, ** *p* < 0.01 versus control. @ *p* < 0.05, non-treated versus MF/RSG-treated group.

C57BL/6J male mice (ranging between 6 and 8 weeks old and body weight between 20 and 22 g) undergoing TBI w/wo chronic TS exposure were used to validate the in vitro data, as detailed in the method section. Body weight and fasting blood glucose levels were regularly measured to assess whether MF and RSG dosing negatively impacted mice’s body weight and glucose levels. As shown in Figure 1, there were no significant differences in body weight within the tested groups at Day 0. However, at the end of the three weeks of TS exposure before TBI, the body weight of animals chronically exposed to TS was slightly lower (yet statistically significant) compared to the controls, which might be due to the appetite suppressant effect as well as a moderate increase in metabolism promoted by TS [49]. The body weight of the animals receiving either MF- or RSG-treated animals following TBI was not statistically different from the TBI controls.

**Figure 2:**
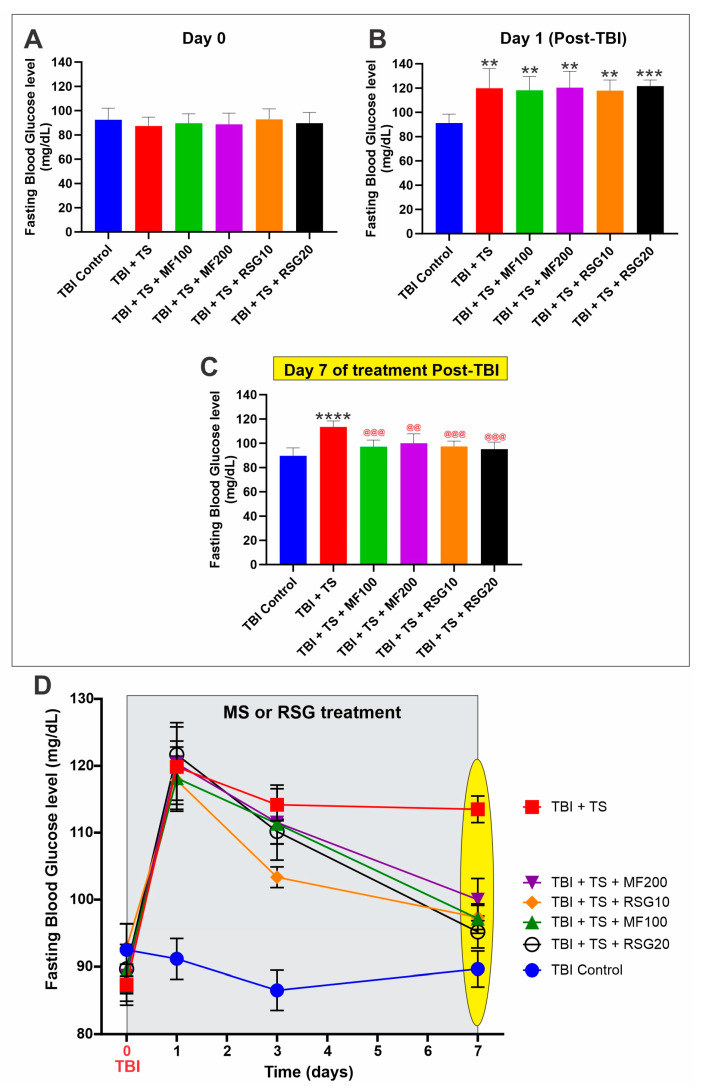
(**A**) Measurements of animals’ fasting blood glucose levels did not significantly differ between the tested groups at Day 0. However, significant differences between controls and test groups were evident from day 1 post-TBI (**B**). By day 7 post-TBI MF and RSG treatment showed to effectively controlled the rising glucose levels of TS-exposed mice (**C**). (**D**) Longitudinal representation of fasting blood glucose levels from time 0 to day 7 post-TBI. *n* = 6 biological replicates, ** *p* < 0.01, *** *p* < 0.001, **** *p* < 0.0001 versus control. @@ *p* < 0.01, @@@ *p* < 0.001, non-treated versus MF/RSG-treated group.

Fasting blood glucose levels across the groups were within limits with no remarkable inter-group differences at Day 0 (Figure 2A) but became significantly more elevated in TS-exposed animals by the end of the three weeks of premorbid smoke conditioning (Figure 2B). This datum is consistent with the higher blood glucose levels reported in smokers. However, by Day 7 post-TBI, the blood glucose levels of the animals receiving either MF or RSG were again comparable to that of the TBI controls (Figure 2C). The longitudinal analysis of fasting blood glucose levels is shown in Figure 2D.

### 2.2. MF and RSG Upregulate NRF2 and Its Downstream Effector Molecules

**Figure 3:**
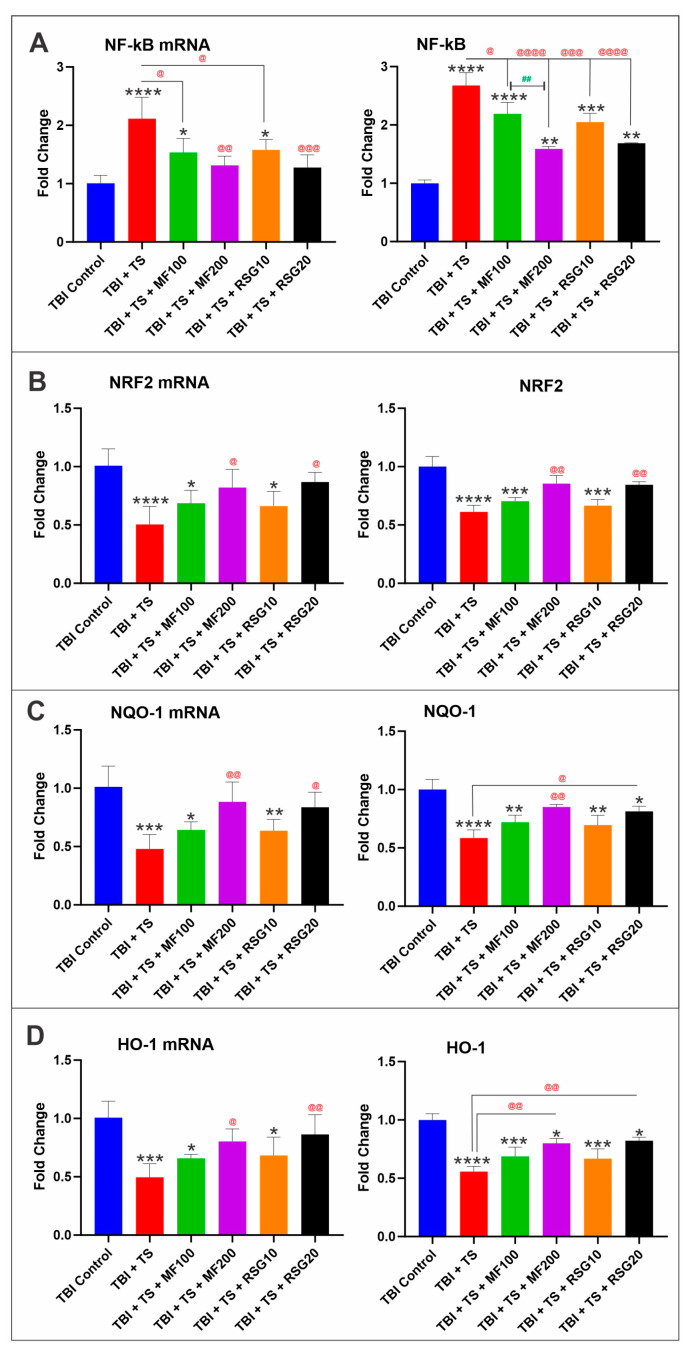
Dose-dependent protective effects of MF and RSG on the antioxidative response system in vivo. ELISA and quantitative RT-PCR analyses emphasized the antioxidative effects of MF and RSG on (**A**) downregulation of NF-kB and (**B**) activation of NRF2 expression levels in a dose-dependent manner. Changes in NRF2 expression levels were paralleled by corresponding variations in the expression levels of its downstream detoxifying effector molecules (**C**) NQO-1 and (**D**) HO-1. *n* = 6 biological replicates, * *p* < 0.05, ** *p* < 0.01, *** *p* < 0.001, **** *p* < 0.0001 versus control. @ *p* < 0.05, @@ *p* < 0.01, @@@ *p* < 0.001, @@@@ *p* < 0.0001 non-treated versus MF/RSG-treated group. ## *p* < 0.01MF100 versus MF200.

MF and RSG have been reported to exert their antioxidative response by promoting the activation of the nuclear factor erythroid 2-related factor 2 (NRF2), which in turn upregulates the expression of its downstream detoxifying effector molecules Heme oxygenase 1 (HO-1) and NAD(P)H dehydrogenase [quinone] 1 (NQO-1) [48,49,50] and downregulates nuclear factor kappa B (NF-κB). To assess if MF and RSG were acting through the same pathway in the context of TS and TBI, we evaluated the protein expression and respective mRNA levels of NF-kB, NRF2, HO-1, and NQO-1 through ELISA and quantitative RT-PCR analyses. Consistent with our previous results, chronic TS exposure significantly upregulated the expression level of NF-kB (an essential modulator and inducer of inflammatory activities; see Figure 3A) and downregulated NRF2 expression (Figure 3B) and its downstream effectors, including HO-1 (Figure 3C) and NQO-1 (Figure 3D). Our results show that MF and RSG reduced NF-κB upregulation driven by TS and increased Nrf2 expression along with its downstream effectors compared to their non-treated counterparts.

### 2.3. MF and RSG Reduce OS and Inflammatory Responses Enhanced by Premorbid TS Exposure

**Figure 4:**
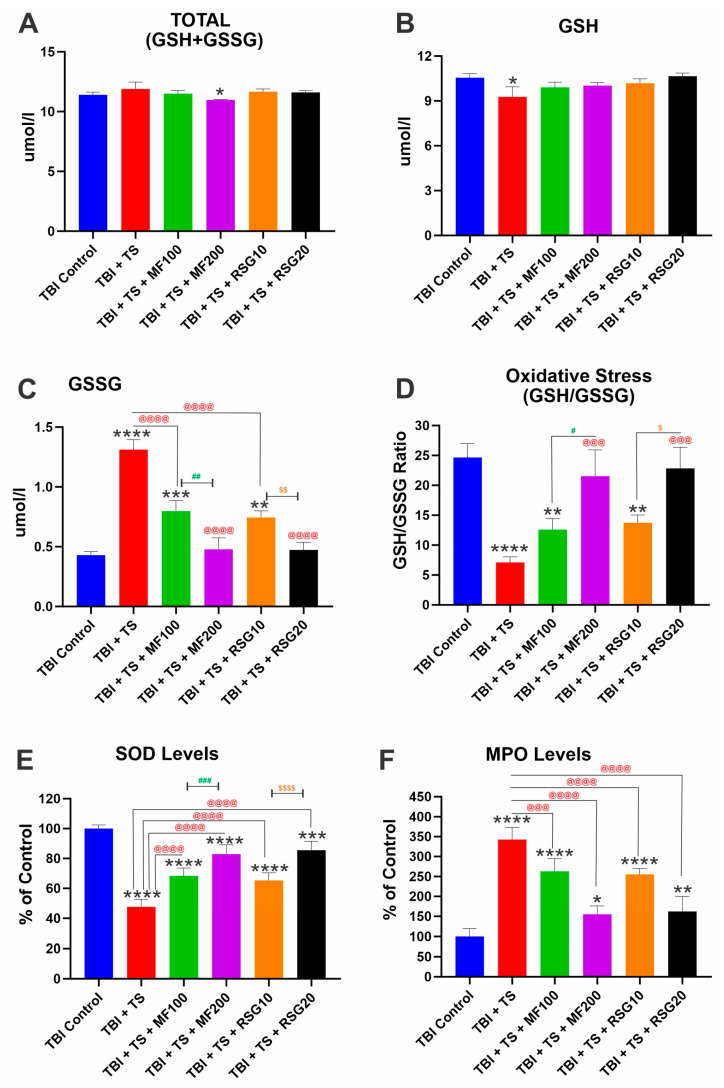
MF and RSG affect OS generated after premorbid TS exposure and TBI induction of mice. (**A**) total glutathione (GSH+ GSSG), (**B**) reduced glutathione (GSH), (**C**) oxidized glutathione (GSSG), (**D**) GSH/GSSG, (**E**) SOD levels, and (**F**) MPO levels. *n* = 6 biological replicates, * *p* < 0.05, ** *p* < 0.01, *** *p* < 0.001, **** *p* < 0.0001 versus control. @@@ *p* < 0.001, @@@@ *p* < 0.0001 non-treated versus MF/RSG-treated group. # *p* < 0.05, ## *p* < 0.01, ### *p* < 0.001 MF100 versus MF200. $ *p* < 0.05, $$ *p* < 0.01, $$$$ *p* < 0.0001 RSG10 versus RSG20.

We measured several OS biomarkers, including glutathione, superoxide dismutase (SOD), and myeloperoxidase (MPO). As shown in Figure 4A–F, the total levels of glutathione encompassing its reduced (GSH) and oxidized (GSSG) forms (GSH + GSSG) are unaffected by the experimental conditions (Figure 4A). However, when looking at its specific forms (reduced and oxidized) separately (Figure 4B,C), we determined that TS negatively impacts GSH plasma levels, thus promoting a corresponding increase in GSSG. This is also evident from the calculated GSH/GSSG ratios (Figure 4D). Similarly, TS reduced the levels of SOD (Figure 4E) and MPO (Figure 4F) compared to the corresponding TBI controls (6.17 U/mL and 16.8 ng/mL, respectively). In each case, treatment with MF or RSG counteracted the pro-oxidative impact of TS in a dose-dependent manner (see Figure 4E,F).

**Figure 5:**
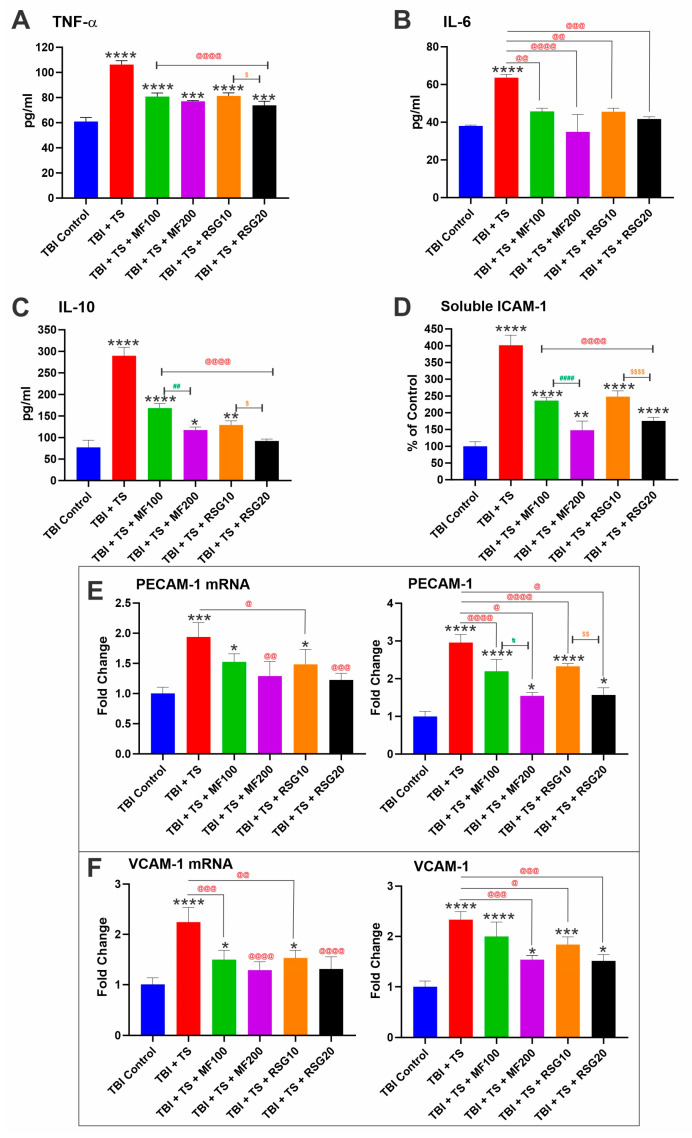
MF and RSG decrease inflammatory responses induced by premorbid TS exposure and TBI induction. ELISA results of pro-inflammatory cytokines (**A**) TNF-α, (**B**) IL-6, (**C**) IL-10, and (**D**) soluble ICAM-1 7 days after TBI. Protein expression and mRNA levels of the inflammatory adhesion molecules (**E**) PECAM-1 and (**F**) VCAM-1 were upregulated by TS exposure and TBI induction. *n* = 6 biological replicates, * *p* < 0.05, ** *p* < 0.01, *** *p* < 0.001, **** *p* < 0.0001 versus control. @ *p* < 0.05, @@ *p* < 0.01, @@@ *p* < 0.01, @@@@ *p* < 0.0001 non-treated versus MF/RSG-treated group. # *p* < 0.05, ## *p* < 0.01, #### *p* < 0.0001 MF100 versus MF200. $ *p* < 0.05, $$ *p* < 0.01, $$$$ *p* < 0.0001 RSG10 versus RSG20.

Inflammatory cytokine expression plays a key role in the inflammatory cascade reactions and neuronal damage after TBI. To determine the influence of MF and RSG on neuroinflammation following TS exposure and TBI, inflammatory cytokines, including TNF-α, IL-6, and IL-10, were evaluated in vivo by ELISA. As shown in Figure 5A–C, the expression levels of these key pro-inflammatory and that of soluble ICAM-1 (Figure 5D) were significantly upregulated in TBI mice exposed to TS compared to TBI controls. It is also evident how MF and RSG treatments reduced the pro-inflammatory effect of TS. Measurements were performed in mice’s plasma samples collected 7 days post-TBI.

Furthermore, we used a combination of ELISA and quantitative RT-PCR to measure changes in the expression levels of inflammatory adhesion molecules, including Platelet Endothelial Cell Adhesion Molecule 1 (PECAM-1) and Vascular Cell Adhesion Protein 1 (VCAM-1). We observed a remarkable increase in protein expression and mRNA levels of PECAM-1 (Figure 5E) and VCAM-1 (Figure 5F) in mice exposed to TS and subjected to TBI. Moreover, in this case, MF and RSG reduced the inflammatory response elicited by TS on TBI-induced mice, and the effect of the treatments was seemingly dose-dependent.

### 2.4. MF and RSG Reduced the Negative Impact of Chronic Premorbid TS Exposure on BBB Disruption by TBI

**Figure 6:**
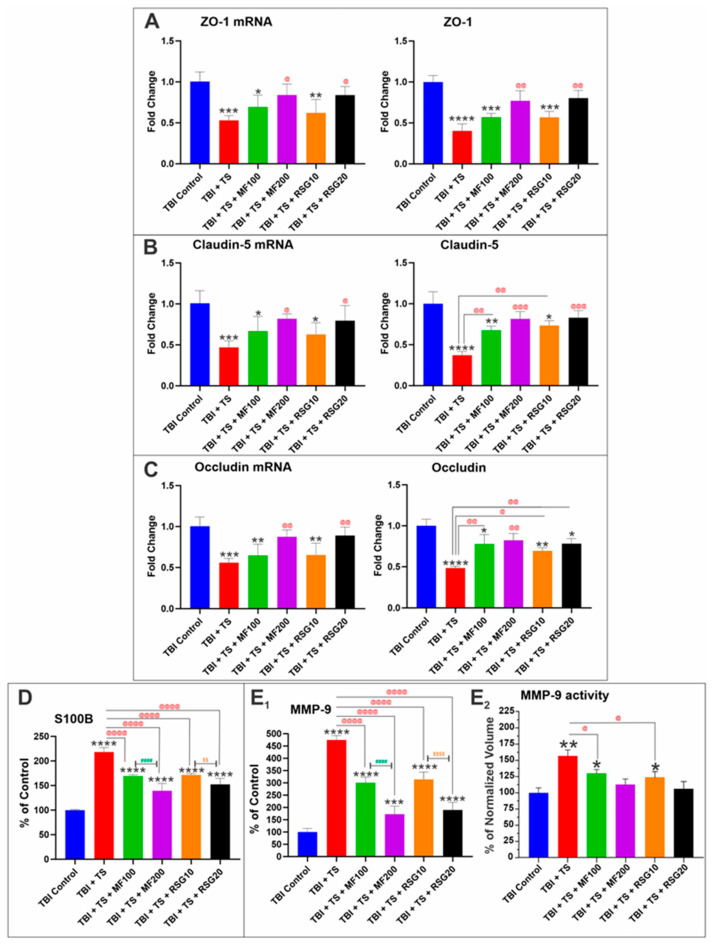
(**E_1_**) MMP-9 expression and (**E_2_**) enzymatic activity. ELISA and quantitative RT-PCR analyses demonstrated upregulation of (**A**) accessory anchoring protein ZO-1 and TJ proteins, (**B**) Claudin-5, and (**C**) Occludin in MF and RSG-treated mice in a dose-dependent manner. Decreased levels of (**D**) S100B and (**E1**) MMP-9 expression and (**E2**) enzymatic activity as biomarkers of BBB integrity emphasized the dose-dependent protective effect of MF and RSG in mice exposed to TS and induced by TBI. *n* = 6 biological replicates, * *p* < 0.05, ** *p* < 0.01, *** *p* < 0.001, **** *p* < 0.0001 versus control. @ *p* < 0.05, @@ *p* < 0.01, @@@ *p* < 0.01, @@@@ *p* < 0.0001 non-treated versus MF/RSG-treated group. #### *p* < 0.0001 MF100 versus MF200. $$ *p* < 0.01, $$$$ *p* < 0.0001 RSG10 versus RSG20.

We have previously shown that changes in Nrf2 expression/activity are paralleled by similar alterations in tight junction (TJ) expression, which can affect the integrity of the BBB [35,41,51]. Consistent with their effect on Nrf2 expression/activity, our analyses of brain tissue homogenates from the corresponding mice groups showed that TS exposure plus TBI induction significantly decreased the protein expression and the corresponding mRNA levels of ZO-1 and TJ proteins, Claudin-5 and Occludin (Figure 6A–C), compared to the TBI controls. The effect was a heightened loss of BBB integrity, which was outlined by the significantly higher level of S100β (a marker of BBB integrity and TBI injury [52,53]) extravasating from the brain into the blood circulation (Figure 6D). In addition, we observed an upregulation and increased activity of MMP-9, which is responsible for the extracellular matrix degradation of the BBB and thus the pathophysiology of BBB breakdown after TBI [54,55,56]) (see Figure 6E_1_,E_2_). S100β and MMP-9 levels were measured in collected plasma samples 7 days post-TBI (average MMP-9 and S100B levels in TBI control: 61.14 ng/mL and 1.01 ng/mL, respectively). The negative impact of chronic TS exposure on BBB damage by TBI was dampened in a seemingly dose-dependent fashion by post-injury MF and RSG treatments.

### 2.5. Downregulation of Thrombomodulin and UCH-L1 Prompted by Chronic TS Exposure in TBI-Induced Mice Is Reduced by MF and RSG

**Figure 7:**
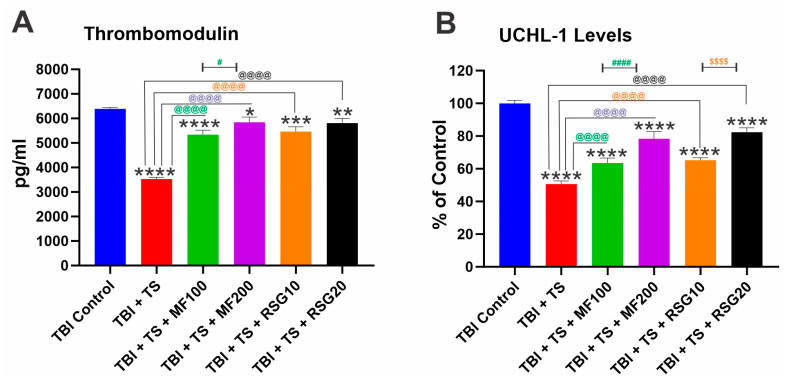
MF and RSG recover the reduced plasma level of Thrombomodulin and UCH-L1 in premorbid TS-exposed and TBI-induced mice. ELISA measurement of (**A**) UCH-L1 and (**B**) Thrombomodulin levels in the blood samples collected 7 days after TBI. *n* = 6 biological replicates, * *p* < 0.05, ** *p* < 0.01, *** *p* < 0.001, **** *p* < 0.0001 versus control. @@@@ *p* < 0.0001 non-treated versus MF/RSG-treated group. # *p* < 0.05, #### *p* < 0.0001 MF100 versus MF200. $$$$ *p* < 0.0001 RSG10 versus RSG20.

As shown in Figure 7A, chronic TS exposure reduced the expression level of the anticoagulant factor thrombomodulin. This datum suggests that TS can potentially hamper the endothelial response to TBI and enhance the risk of blood coagulation in TBI patients, as previously shown [57,58]. However, TS-induced downregulation of thrombomodulin was effectively negated by MF and RSG (Figure 7A). Furthermore, analyses of plasma samples of TS-exposed and TBI-induced mice also revealed a reduction in ubiquitin C-terminal hydrolase L1 (UCH-L1) levels. UCH-L1 is thought to play a key role in maintaining axonal integrity [59], thus providing neuroprotection. Interestingly, MF and RSG reduced the negative impact of TS on Thrombomodulin and UCH-L1 in a dose-dependent manner (Figure 7A,B) (average UCH-L1 level in TBI control: 16.01 ng/mL).

### 2.6. MF and RSG Decrease the Negative Effect of Chronic TS Exposure on the Loss of Motor Activity and Recovery Post-TBI

**Figure 8:**
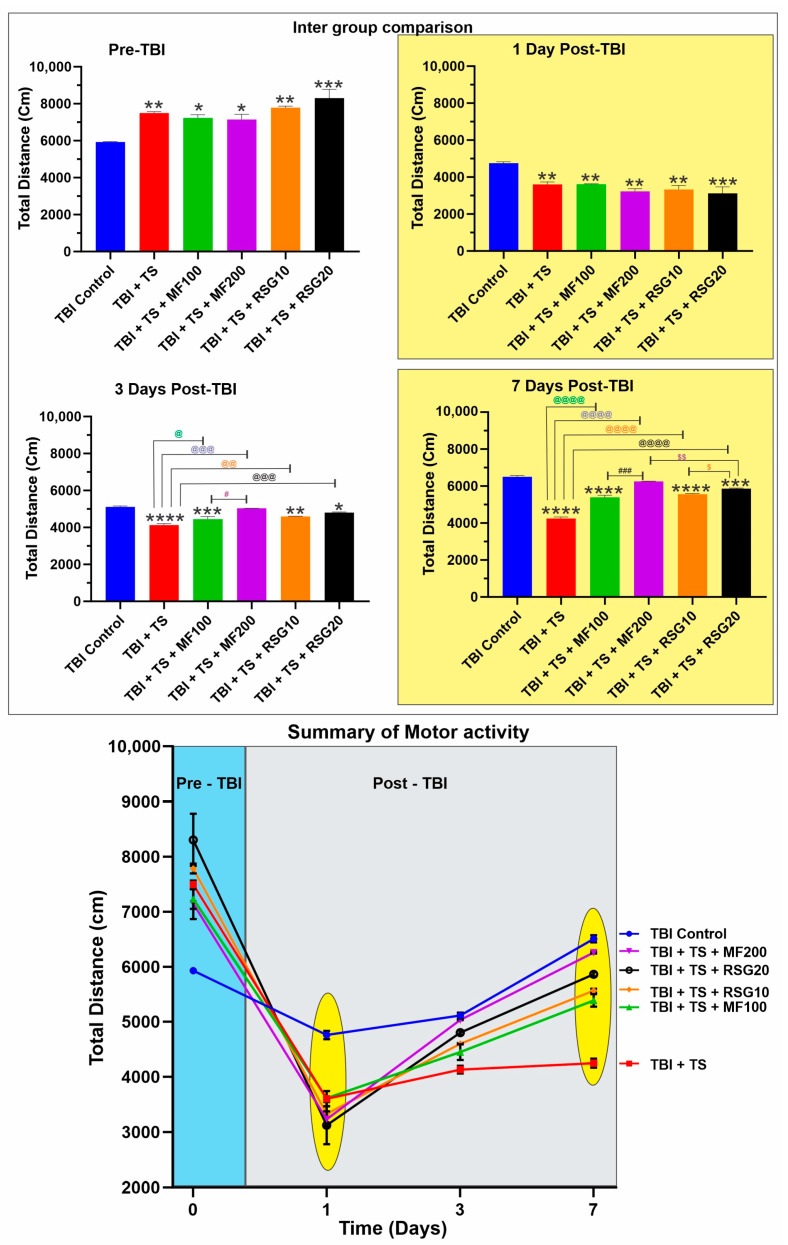
Effect of MF and RSG on exploratory behavior and general motor activity after premorbid TS exposure and TBI induction of mice. Mice undergoing chronic TS exposure demonstrated significantly higher motor activity. Measurements performed at 24 h, 3 days, and 7 days post-TBI showed that TS further aggravated TBI injury as denoted by the significant reduction in motor activity compared to the control. However, an assessment of the mice’s response to TBI and TS exposure w/wo MF and RSG showed that treated mice experienced a recovery in motor activity compared to non-treated animals. *n* = 6 biological replicates, * *p* < 0.05, ** *p* < 0.01, *** *p* < 0.001, **** *p* < 0.0001 versus control. @ *p* < 0.05, @@ *p* < 0.01, @@@ *p* < 0.01, @@@@ *p* < 0.0001 non-treated versus MF/RSG-treated group. # *p* < 0.05, ### *p* < 0.001 MF100 versus MF200. $ *p* < 0.05, $$ *p* < 0.01 RSG10 versus RSG20.

Exploratory behavior and general motor activity of the test mice were regularly recorded to assess the therapeutic effect of MF and RSG administration on the TBI outcomes after premorbid TS exposure. As shown in Figure 8, mice chronically exposed to TS demonstrated significantly higher motor activity, which is evident from the total distance traveled by the animals after being subjected to chronic TS exposure for three weeks. This datum is consistent with the metabolic effect of TS and a craving for TS. Measurements performed at 1, 3, and 7 days post-TBI revealed that TS exposure significantly aggravated TBI-induced neurological impairment, as denoted by the remarkable reduction in motor activity compared to the TBI controls. However, assessment of the mice’s response to TBI and TS exposure w/wo MF and RSG clearly showed that treated mice experienced a recovery of motor activity beginning at day 3 of the post-TBI drug treatment, which further improved on day 7 post-TBI following the daily administration of the drugs. The positive effect of MF and RSG on the recovery of motor activity was dose-dependent (see also the longitudinal pattern of motor activity). From an exploratory behavioral perspective, rodents typically prefer not to station at the center of the apparatus and tend to walk close to the walls (thigmotaxis) since this is a novel environment for them. As shown in Supplementary Figure S1A, all animals before TBI exhibited a similar exploratory pattern where mice chronically exposed to TS traveled more distance (see Figure 8) with the most movements and very low resting time. However, the walking pattern 24 h post-TBI showed a significant reduction in the traveled distance, movements, and an extended resting time, which is remarkably clear in Figure S1B in the untreated TS-exposed mice. The TS-exposed animals receiving MF and/or RSG showed a more uniform walking pattern across the chamber perimeter with reduced resting time than the untreated TS-exposed animals. The walking pattern performed at 3 days and 7 days post-TBI continued to show more traveled distance and movements and less resting time in MF and RSG-treated animals than TS (see Figure S1C,D), suggesting a faster recovery of motor activity than in non-treated animals.

## 3. Discussion

Several pathophysiological mechanisms and prodromal factors involved in the onset of traumatic and post-traumatic brain injuries have been elucidated [3]; however, the complexity of these mechanistic pathways continues to pose a severe drag to the development of more effective therapeutic strategies. It was recently shown that premorbid conditions such as chronic smoking negatively impact TBI severity and post-TBI recovery. TS contains over 4700 toxic compounds, including nicotine and ROS, which not only trigger local OS damage and inflammation in the lungs but can initiate OS and inflammation in other tissues remote from the primary exposure site [33], including the cerebrovascular system, thus promoting endothelial dysfunction, particularly at the level of the BBB [60,61].

Moreover, TS can trigger BBB disruption via oxidative and inflammatory mechanisms [62,63]. It is conceivable that TS synergistically influences TBI by exacerbating BBB disruption and promoting OS and neuroinflammation. Enhanced ROS production and OS have been implicated in the onset and pathogenesis of numerous neurodegenerative and cerebrovascular disorders [64]. In this respect, NRF2 is a transcription factor that regulates the cellular response to OS, increasing cytoprotective enzyme expression. Because of the cross-talk between the antioxidant and anti-inflammatory pathways, many of the anti-inflammatory and mitochondrial actions of NRF2 have been considered secondary to its antioxidant impacts. Emerging evidence recently confirmed that the knockout (KO) of NRF2 aggravates TBI pathology, while NRF2 activators inhibit neuronal apoptosis and neuroinflammatory responses by alleviating oxidative damage [2]. Unsurprisingly, there is a rising interest in investigating the association between the impairment of NRF2 signaling and TBI, thus the mounting research interest in identifying new therapeutic potential targeting NRF2 to prevent and/or reduce brain injury.

As a first-line biguanide class of oral antidiabetic drugs, MF has been shown to have anti-inflammatory, anti-cancer, cardioprotective, hepatoprotective, and antioxidant effects. Currently, it is being investigated as a drug directly acting on the CNS [43,65,66,67,68]. MF can easily cross the BBB, accumulate in the mitochondria, and activate the NRF2 antioxidant pathway, which eventually enhances the expression of antioxidant enzyme genes such as SOD, Glutathione peroxidase 1 (Gpx1), and catalase (CAT) [69]. With its mild side effects, safe pharmacokinetic profile, and multi-functional properties, MF has become a favorable neurotherapeutic agent for repurposing [46]. Like MF, RSG, a PPARγ agonist and a member of the thiazolidinedione family, has been known to have strong anti-inflammatory and antioxidant properties [50,70]. RSG directly affects cellular function by inducing PPARγ, which regulates the transcriptional activity and the expression of downstream target genes. RSG reduces/prevents ROS production and its consequent damaging effect by regulating detoxification enzymes (CAT and SOD) and ROS scavengers [71]. RSG is known to have a lower safety profile than MF, and the FDA has restricted its use following studies that linked the drug to an increased risk of heart attacks. Thus, unlike MF, using RSG cannot be considered a viable long-term preventive strategy. However, RSG possesses significant anti-inflammatory properties on top of its NRF2-enhancing activity, thus providing a feasible post-TBI treatment option over a short period.

The major objective of this study was to determine the effectiveness of MF and RSG in preventing/reducing the impact of chronic TS exposure on the post-TBI loss of BBB integrity and assess the putative mechanism of action and biological targets involved in the process. The premise for this study builds upon our previous works suggesting that MF and RSG attenuate the cerebrovascular impairments and mortality associated with chronic TS. Through those works, the involvement of common pathogenic modulators of BBB impairment involving angiogenic and inflammatory factors released by BBB endothelial cells in response to hyperglycemia (HG) and exposure to TS was proven [51]. Our previous in vitro data showed that RSG and MF could prevent/reduce BBB impairment and OS in response to chronic TS exposure, thus suggesting that these drugs could protect the cerebrovascular system from exogenous oxidative stimuli [49,50]. Our previous in vivo data also showed the protective effect of RSG (10 or 20 mg/kg) and MF (100 or 200 mg/kg) in a dose-dependent manner against TS-induced cerebrovascular toxicity in a rodent model of chronic cigarette smoking [48,49].

To evaluate the neuroprotective effects of MF and RSG to reduce exacerbation of TBI injury by premorbid chronic smoking, we used chronically TS-exposed C57BL/6J male mice subjected to TBI by weight drop [72]. We then performed a set of behavioral and locomotor assessments and examined a panel of brain and blood-based biomarkers encompassing BBB integrity, neuroinflammation, OS, blood hemostasis, and redox metabolism related to the NRF2-ARE signaling pathway.

Recently emerging evidence indicated that OS plays an essential role in the development and pathogenesis of TBI [73,74]. Many superoxide radicals are generated immediately in brain microvessels after injury and increase afterward due to the infiltration of neutrophils and macrophages and the overactivation of microglia [2]. On the other hand, scavengers of oxygen radicals, including SOD and catalase, remarkably reduce the level of superoxide radicals and partly reverse the injury of the brain [75]. Therefore, targeting the suppression of OS in the brain appears as a promising strategy to reduce the burden of chronic smoking on TBI outcomes and ameliorate post-TBI recovery. Our in vivo data show that MF and RSG produced a significant upregulation of NRF2 and its main effectors, NQO-1 and HO-1, suppressed by chronic exposure to TS and dampened the expression of NF-қB, which is the main pro-inflammatory modulator (see Figure 3A). This is consistent with previous reports by our group [35,49] and additional studies by others suggesting that metformin administration inhibits microglia activation-mediated inflammation via NF-қB and the MAPK signaling pathway [47,49], thus improving neurobehavioral function following TBI, as we also observed in this study. RSG promotes not only Nrf2 upregulation but also the activation of the transcription factor peroxisome proliferator-activated receptor gamma (PPAR-γ) [48]. PPAR-γ activation has been shown to inhibit neurotoxicity in mice after TBI [76], prevent microglial polarization/activation [77], and reduce autophagy in experimental traumatic spinal cord injury [78]. Thus, as a strong PPAR-γ agonist, RSG effectively reduces inflammation, alleviates free radical generation and OS-induced tissue damage, and promotes neurological recovery [79].

The BBB physically separates most brain regions from the peripheral circulation to limit the entry of neurotoxic substances from the periphery into the CNS and maintain structural and functional homeostasis in the brain [80,81]. Complex TJ proteins, including Occludin and Claudins, tightly connect the endothelial cells forming the vascular layer of the BBB. Several studies have reported TS-induced alterations of TJ protein expression and distribution, resulting in a loss of BBB integrity, ionic imbalance within the neurovascular unit (NVU), and intracellular ROS followed by the onset of inflammation [82]. BBB disruption is also a major pathophysiological feature of TBI and is similarly characterized by TJ disruption, enhanced paracellular permeability, and inflammation [83]. Furthermore, unopposed neuroinflammation can promote the loss of BBB integrity, creating a vicious cycle [27].

Concerning the effect of premorbid chronic smoking on the impact of TBI on the BBB, our data clearly showed that the expression of TJ proteins such as Clauding-5, Occludin, and the TJ accessory protein ZO1 are significantly downregulated in TBI mice that were chronically exposed to TS compared to their control counterparts (Figure 6A–C). Noticeable too is the fact that post-TBI treatment with either MF or RSG promoted a significant TJ upregulation suggesting a partial restoration of BBB integrity which was also confirmed by the reduced BBB extravasation of S100β (Figure 6D), a known marker of BBB integrity and TBI injury [52,53,84,85]. The same trend was observed when we assessed the plasma levels and activity of MMP-9 (Figure 6(E_1_,E_2_)), an inflammation biomarker that regulates vascular remodeling through extracellular matrix degradation [86]. It has been proven that inflammatory cytokines are directly linked to increased MMP-9 levels [87] and concur with BBB dysfunction [88]. Previous studies by our group have shown that Nrf2 downregulation by small inhibitor RNA promoted a downregulation of TJ and adherens junctions proteins such as Claudin-5 and vascular endothelial Cadherin, ultimately resulting in a loss of BBB integrity and enhanced permeability to polar molecules [41]. Our study results are in line with these previous findings where counteracting Nrf2 downregulation enhanced by TS exposure with MF or RSG treatments resulted in less significant losses of BBB integrity post-TBI as assessed by measurements of S100β extravasation.

Neuroinflammation is a key etiological factor in the onset of primary brain injury following TBI; thus, reducing the inflammatory response post-TBI can lessen brain damage [21,36,37]. After TBI, a robust inflammatory response occurs acutely, characterized by the activation of resident cells, the migration and recruitment of peripheral immune cells, and the release of inflammatory mediators [89]. This set of events is put in motion by the pro-inflammatory transcription factor NF-κB, which regulates multiple aspects of innate and adaptive immune functions and is a pivotal mediator of inflammatory responses [90]. NF-κB triggers the cascade amplification of inflammatory responses, inducing secondary brain damage after TBI [91]. Indeed, inhibiting NF-κB-mediated neuroinflammatory response has been shown to facilitate post-TBI recovery [92].

Interestingly, the NRF2 and NF-κB pathways interfere with each other at their corresponding transcription level [88]. NRF2 suppresses the activation of the NF-κB pathway by promoting antioxidant defenses, thus reducing ROS-mediated NF-κB activation. By contrast, NF-κB can suppress NRF2 activity, thus preventing ARE gene transcription [88]. Our in vivo results show that chronic TS exposure decreases NRF2 expression (and its corresponding effectors, including NQO-1 and HO-1), leading to the upregulation of NF-kB (see Figure 3A), resulting in increased inflammation. This is evidenced by the increased pro-inflammatory biomarkers, including adhesive molecules and inflammatory cytokines (see Figure 5A–F). Interestingly, RSG and MF prevented or significantly reduced the dampening effect of chronic TS exposure on Nrf2 levels and corresponding effectors (see Figure 3), resulting in a less severe inflammatory response in treated animals compared to their untreated counterparts (Figure 5).

The effect of MF and RSG treatments on the renormalization of Nrf2 expression is also well evidenced by the reduction in OS generated in the premorbid TS-exposed animals after TBI induction compared to their untreated counterparts (Figure 4A–F). Our data show that chronic TS exposure decreases Nrf2 leading to a notable increase in ROS levels and a decrease in UCH-L1 (see Figure 7B). UCH-L1 is a biomarker of TBI severity [93]; it is highly expressed in neurons and is a major target of OS where increased ROS induces oxidative modification of UCH-L1, leading to pathological alteration of their cellular function [94,95]. Noteworthy is the fact that MF and RSG significantly dampened UCH-L1 downregulation promoted by TS in a dose-dependent fashion. Interestingly, a recent study by Namani et al. [96] provides evidence that Nrf2 directly binds with the promoter regions of UCH-L1 and triggers its expression. Although additional studies will be required to detail the underlying mechanisms, our results are in line with this finding where downregulation of Nrf2 by chronic smoking is paralleled by downregulation of UCH-L1 and the counteractive effects of MF and RSG preventing TS-induced Nrf2 downregulation result in a significantly lesser decline of UCH-L1 when compared to the untreated animals. In addition, recovery of motor activities post-TBI (see Figure 8 and Figure S1) was significantly improved, which seems in line with the side-by-side histopathological assessment of brain tissue damage seven days post-TBI (see Figure S2) from a sub-cohort of animals from the main four test groups corresponding to TBI, TBI + TS, TBI + TS + MF200 (highest dose tested), and TBI + TS + RSG20 (highest dose tested) and the controls. In addition, studies by others also suggest that activation of Nrf2 and/or PPAR-γ afford neuroprotection and neurological recovery.

MF and RSG can negatively affect glycemia by being antidiabetic drugs. However, chronic TS exposure is a prodromal factor to the onset of type-2 diabetes by inducing insulin resistance, hence increased glycemia, in which case MF and RSG would provide additional benefit.

Limitation of the current study: Recently published data have shown that IP injection of MF and RSG did not affect the body weight of treated mice and fasting blood glucose levels across the experimental groups. Moreover, although IP injection is not a common clinical route of administration, this route was chosen because of the practical difficulties of doing repetitive IV injections or repetitive delivery of MF and RSG in drinking water in mice and an understanding that there should not be a big difference in the pharmacokinetic profiles of a small molecule upon IV vs. IP administrations in mice [97,98]. Overall, efficacy and BBB permeability experiments utilizing systemic routes of administration should translate well to future oral administration. Furthermore, we used a male population of C57BL/6J mice chronically exposed to TS. Due to this application’s limited scope and time, a more comprehensive investigation involving both genders will be deferred later. We also plan to include a mixed (male and female) population of Nrf2 KO (Nfe2l2tm1Ywk/J) mice to further validate the relevance of Nrf2 in the neurovascular protection afforded by MS and RSG.

MF and RSG treatments were limited to post-TBI only. The main reason is that RSG has a much lower safety profile than MF. Therefore, unlike MF, RSG is not indicated for long-term preventive treatments but could be a valid therapeutic strategy in the short term if used, as in this case, to reduce the burden of TBI. We have previously shown that MF can effectively reduce the negative impact of chronic smoking on stroke outcomes and secondary brain injury [35], and we plan to assess its effectiveness in a TBI setting in upcoming studies. The biological effect of the drug treatments (in the specific case, oxidative stress reduction linked to upregulation/restoration of Nrf2 activity) was limited to protein expression analyses and RT-PCR. The main reasons we took this approach at this time instead of going to a more extended set of analyses are two folds: (1) The manuscript focus was primarily on assessing whether these antidiabetic drugs may have a repurposed use in reducing the impact of TS in the progression and/or outcome of neurological disorders such as TBI rather than focusing on their biological activities. (2) We have already investigated (and published quite extensively) the underlying mechanisms correlating the biological effects of these two drugs to Nrf2, NF-κB, oxidative stress, BBB integrity, etc. [35,41,48,49,50,52,88,99,100,101,102]. In this respect, we felt that repeating more mechanistic confirmatory experiments was unnecessary and/or would not add to the already available bulk of data on the subject.

## 4. Materials and Methods

### 4.1. Reagents and Materials

Reagents and chemicals were purchased from Sigma-Aldrich (St. Louis, MO, USA) or Bio-Rad Laboratories (Hercules, CA, USA). Quantikine ELISA kits were obtained from R & D systems (Minneapolis, MN, USA), Thermo Fisher Scientific (Waltham, CA, USA), and MyBioSource (San Diego, CA, USA). Additional ELISA kits were purchased from Aviva Systems Biology (San Diego, CA, USA). Pierce BCA Protein Assay Kit (#23225) was purchased from Thermo Fisher Scientific. Gel electrophoresis was performed using Mini-Protean^®^TGXTM gels 4–15% (#456–1084) from Bio-Rad Laboratories. Rosiglitazone (RSG # A00183, MW: 357.4) was obtained from Adipogen, and metformin (MF #PHR1084, MW:165.6) was obtained from Sigma-Aldrich.

### 4.2. Experimental Design

In this study, all experimental procedures performed on the mice were approved by the Institutional Animal Care and Use Committees (IACUC), OU, Rochester, MI, USA. Thirty-Six C57BL/6J male mice (ranging between 6 and 8 weeks old and body weight between 20 and 22 g) were purchased from Jackson Laboratory and group-housed in a temperature-controlled environment under a 12/12-h light/dark cycle with free access to food and water. Mice were divided into six major groups (6 mice/group), including TBI Control, TBI + TS, TBI + TS + MF100, TBI + TS + MF200, TBI + TS + RSG10, and TBI + TS + RSG20 (see also Table 1).

Mice were given one-week post-arrival for acclimatization in the new housing for recovery upon transport. All the groups, except the TBI controls, were chronically and simultaneously exposed to sidestream smoke derived from 3R4F standardized research cigarettes via direct inhalation. Sidestream smoke was generated using a Single Cigarette Smoking Machine (SCSM, CH Technologies Inc., Westwood, NJ, USA) following previously published methods [35] (see also Figure S3). Mice were exposed to TS mixed with oxygenated air six times/day; two cigarettes/hour, 6–8 h/day, seven days/week for a total of three weeks, according to the ISO/FTC standard smoking protocol, as mentioned earlier.

### 4.3. Induction of Head Injury in Mice

TBI was induced by a standard weight-drop procedure [103,104]. This model mimics human head injury by using a standardized weight-drop device inducing a focal blunt trauma over an intact skull without pre-injury manipulations. The impact stimulates a robust neuroinflammatory response, BBB breakdown, and neurological impairment. In brief, anesthetized mice were placed on a spongy surface under the weight-drop device. Head movements were allowed parallel to the injury plane at the induction time to mimic a mild-to-moderate head injury. During the induction phase, mice were positioned to direct the trauma from the left anterior frontal area at the same distance between the eye and the ear. Then a metal weight of 30 g was allowed to free fall from 80 cm above the head through a vertical hollow tube (with an internal diameter of 13 mm) as the guiding system (see also Figure S3 step 2). The sham-injured mice underwent the same procedures, excluding being subjected to head injury by weight drop.

### 4.4. Rosiglitazone and Metformin Treatment In Vivo

Mice were treated with RSG or MF following TBI (no pre-TBI treatment). Drugs were dissolved in DMSO/sterile saline (1:10) and sterile saline, respectively, and administered daily via intraperitoneal (IP) injections for final dose levels of 10 or 20 mg/kg for RSG and 100 or 200 mg/kg for MF with final dose volume of 20 mL/kg for a consecutive 7 days after TBI [70].

### 4.5. Fasting Blood Glucose Level Analysis

A tail pinprick was conducted on the anesthetized mice to determine the mice’s fasting blood glucose levels. The glucose level was measured using a contour next blood glucose meter obtained from Bayer Healthcare (Indianapolis, IN, USA).

### 4.6. Open Field Test

As a standard measure of exploratory behavior and general activity in rodents, open field tests were performed using a SuperFlex system (Omnitech Electronics, Columbus, OH, USA) [105]. Briefly, mice were housed in a 16″ × 16″ acrylic chamber containing infrared photosensors. Then the mice were monitored and recorded for 1 h, and the first 30 min of 1 h were excluded as the acclimatization period (Figure S3 step 3). Fusion Software performed automatic analyses of the mice’s total activity. All behavioral analyses were performed between 9 a.m. and 1 p.m.

### 4.7. Blood Collection and Brain Isolation

Mice were euthanized under terminal anesthesia 7 days after TBI to collect blood and brain samples for subsequent biochemical and molecular analyses. Blood samples were collected by cardiac puncture as described elsewhere [58]. Briefly, mice were positioned on their back and anesthetized with inhaled isoflurane (4% induction; 2% maintenance) to minimize discomfort, distress, and pain. Then a V-cut was made through the skin and abdominal wall, and internal organs were moved to the side. The needle was inserted through the diaphragm and into the heart. Blood was collected by applying negative pressure on the syringe plunger. To isolate the brain, we cut at the nape and then extended along the midline from the dorsal cervical area to the tip of the nose. The skin was then pulled away from the skull laterally. The skull was cut and opened by placing the point of the scissors in the foramen magnum and cutting along the midline. After levering away parietal bones from the brain and disrupting the nerve attachments at the brain stem and the optic chiasm, the brain was removed from the skull, rinsed into sterile, cold PBS, and then frozen in liquid nitrogen and stored at −80 °C [48,58].

### 4.8. Hematoxylin and Eosin (H&E) and Nissl Staining

Seven days post-TBI, a subgroup of mice from each of the main four test groups including TBI, TBI + TS, TBI + TS + MF200 (highest dose tested), TBI + TS + RSG20 (highest dose tested), and controls were euthanized as described above, their brains removed and immediately fixed in 4% PFA at 4 °C for 48 h to prevent possible autolysis. PFA-fixed brain tissues were then embedded in paraffin and shipped in cold storage to iHisto, a histopathology lab support firm (iHisto; Boston, MA, USA) that provides tissue processing, routine histology, and molecular pathology services to perform basic H&E and Nissl staining and microscopy assessments of the samples.

### 4.9. Preparation of Protein Extracts, ELISA, and Zymography

We harvested the total proteins of brain tissues by RIPA lysis buffer based on the manufacturer’s instructions. Total proteins were centrifuged at 14,000× *g* for 30 min. The proteins’ quantification of NF-kB, Nrf2, NQO-1, HO-1, PECAM-1, VCAM-1, and tight junctional proteins ZO-1, Claudin-5, and Occludin from brain tissue homogenate was assessed using enzyme-linked immunosorbent assays (Aviva system biology, San Diego, CA, USA) according to the manufacturer procedures. All samples were analyzed in triplicate. Aliquots of brain tissue lysate were also used to determine matrix metalloproteinase-9 activity in gel zymography (Invitrogen Novex Zymogram Gels, Thermofisher, cat# ZY00100BOX) as described by the manufacturer. Lysis bands were measured densitometrically using Phoretix^TM^ 1D analysis software (Nonlinear USA, Inc., Durham, NC, USA). Optical densities were normalized based on known quantities of the specific standard (MMP-9) loaded along with the experimental samples.

Plasma samples were analyzed using Quantikine ELISA kits to evaluate the levels of thrombomodulin, inflammatory cytokines (including IL-6, TNF-α, and IL-10), SOD, MPO, MMP-9, and soluble ICAM-1 according to the manufacturer’s guidelines.

### 4.10. RNA Extraction and Quantitative Real-Time Polymerase Chain Reaction (RT-PCR)

Quantitative RT-PCR was performed according to the protocol used in our previous work [41,106]. In brief, the total genomic RNA was extracted from brain tissues using RNeasy plus mini kit described in the manufacturer’s guideline (Qiagen Inc, Santa Clarita, CA). Complementary DNA (cDNA) was synthesized using a Hight Capacity RNA-to-cDNA kit (#4387406) obtained from Thermo Fisher Scientific. Gene expression was determined by quantitative RT-PCR using SYBR Green Master Mix (#A25741)-based fluorescence procedure. The primer pairs (see sequences in Table 2) were designed based on PubMed GenBank and synthesized by Integrative DNA Technologies (Coralville, IA, USA). The RNA targets were amplified using a Bio-Rad CFX96 Touch Real-Time PCR detection system. Relative quantification of mRNA expression was measured using the 2^−ΔΔCT^ method. All samples were analyzed in triplicate with normalization to the β- actin.

### 4.11. Glutathione Levels Measurement

Plasma samples collected from mice were analyzed using the Quantification Kit for Oxidized and Reduced Glutathione (Sigma-Aldrich, St. Louis, MO, USA). Specifically, we assessed the levels of oxidized glutathione (GSSG) and reduced glutathione (GSH) according to the manufacturer’s guidelines. Fluorescence intensity was measured via a microplate reader (TECAN-Spark Cyto) at the wavelength of 490 nm for excitation and 520 nm for emission.

### 4.12. Statistical Analysis

All the results were reported as mean ± standard deviation (SD). All statistical analyses were processed using GraphPad Prism 9 Software Inc. (La Jolla, CA, USA) through one-way ANOVA followed by Tukey’s or Dunnett’s test to evaluate the significance of the data. *p* values ≤ 0.05 were considered statistically significant.

## 5. Conclusions

The use of antidiabetic drugs to prevent/reduce TS-promoted cerebrovascular impairment associated with TBI has been marginally assessed. Thus, this is the first study to evaluate their putative protective effectiveness and therapeutic feasibility as post-TBI treatments to reduce brain injury and improve outcomes in chronic smokers where premorbid TS exposure plays a significant negative prodromal role driving the severity of the resulting brain injury and neurological dysfunctions. Our recent data shows that MF and RSG activate counteractive antioxidative and anti-inflammatory mechanisms, drastically reducing TS toxicity and impacting TBI outcomes, including loss of BBB integrity loss, OS damage, and neuroinflammation [107]. Our data suggest that repurposing or “extending” the use of MF and RSG in the setting of traumatic brain injury could be a promising therapeutical strategy for treating TBI in chronic smokers by preventing/reducing BBB damage, diminishing TBI severity, and ameliorating post-TBI recovery. This may provide a shift and an extension of practice paradigms and clinical intervention that can enable a rapid transition to prophylactic and/or therapeutic care for what is largely considered an “at-risk” population.

## Figures and Tables

**Table 1:** In vivo experimental design.

	TBI Control	TBI + TS	TBI + TS + MF100	TBI + TS + MF200	TBI + TS + RSG10	TBI + TS + RSG20
TBI	√	√	√	√	√	√
TS Exposure	-	√	√	√	√	√
MF 100 mg/kg	-	-	√	-	-	-
MF 200 mg/kg	-	-	-	√	-	-
RSG 10 mg/kg	-	-	-	-	√	-
RSG 20 mg/kg	-	-	-	-	-	√

“√” indicates that the procedure or treatment was performed. “-” indicates that the procedure or treatment was not performed.

**Table 2:** Forward and reverse primer sequences (5′ -3′) for quantitative RT-PCR.

Target Gene	Forward	Reverse
NRF2	5′- GGC TCA GCA CCT TGT ATC TT -3′	5′- CAC ATT GCC ATC TCT GGT TTG -3′
NQO-1	5′- GAG AAG AGC CCT GAT TGT ACT G -3′	5′- ACC TCC CAT CCT CTC TTC TT -3′
HO-1	5′- CTC CCT GTG TTT CCT TTC TCT C -3′	5′- GCT GCT GGT TTC AAA GTT CAG -3′
NF-kB	5′- AGA CAT CCT TCC GCA AAC TC -3′	5′- TAG GTC CTT CCT GCC CAT AA -3′
Claudin-5	5′- GGT GAA GTA GGC ACC AAA CT -3′	5′- TTT CTC CAG CTG CCC TTT C -3′
Occludin	5′- CAG CAG CAA TGG TAA CCT AGA G -3′	5′- CAC CTG TCG TGT AGT CTG TTT C -3′
VCAM-1	5′- GAG GGA GAC ACC GTC ATT ATC -3′	5′- CGA GCC ATC CAC AGA CTT TA -3′
PECAM-1	5′- CAA CAG AGC CAG CAG TAT GA -3′	5′- TGA CAA CCA CCG CAA TGA -3′
ZO-1	5′- CAT TAC GAC CCT GAA GAG GAT G -3′	5′- AGC AGG AAG ATG TGC AGA AG -3′
Β-Actin	5′- GAG GTA TCC TGA CCC TGA AGT A -3′	5′- CAC ACG CAG CTC ATT GTA GA -3′

## Data Availability

The data presented in this study are available on request from the corresponding author.

## Supplementary Materials

The following supporting information can be downloaded at: https://www.mdpi.com/article/10.3390/ijms24076219/s1.

## Abbreviations

ARE	Antioxidative Response Element
BBB	Blood–Brain Barrier
CNS	Central Nervous System
ELISA	Enzyme-Linked Immunosorbent Assay
HO-1	Heme Oxygenase 1
H&E	Hematoxylin and Eosin
ICAM-1	Intercellular Adhesion Molecule-1
KEAP1	Kelch-Like ECH Associated Protein 1
mBMEC	Mouse Brain Microvascular Endothelial Cells
MF	Metformin
MMP-9	Matrix Metalloproteinase-9
MPO	Myeloperoxidase
NF-κB	Nuclear Factor kappa B
NQO-1	NAD(P)H: Quinone reductase I
NRF2	Nuclear Factor erythroid-2 related Factor 2
OS	Oxidative Stress
PECAM-1	Platelet Endothelial Cell Adhesion Molecule 1
PPARγ	Peroxisome Proliferator-Activated Receptor
ROS	Reactive Oxygen Species
RSG	Rosiglitazone
RT-PCR	Real-Time Polymerase Chain Reaction
SOD	Superoxide Dismutase
TBI	Traumatic Brain Injury
TJ	Tight Junction
TS	Tobacco Smoking
UCH-L1	Ubiquitin C-terminal Hydrolase L1
VCAM-1	Vascular Cell Adhesion Protein 1
ZO-1	Zonulae Occludentes-1
