# Supplementary material for: Antidiabetic Drugs Can Reduce the Harmful Impact of Chronic Smoking on Post-Traumatic Brain Injuries
Source: Int J Mol Sci. 2023 Mar 25;24(7):6219. doi: 10.3390/ijms24076219 (PMC10093862; doi:10.3390/ijms24076219)
Supplement: Supplementary file 1 [file ijms-24-06219-s001.zip › ijms-2291755-supplementary.pdf]

International Journal of
Molecular Sciences

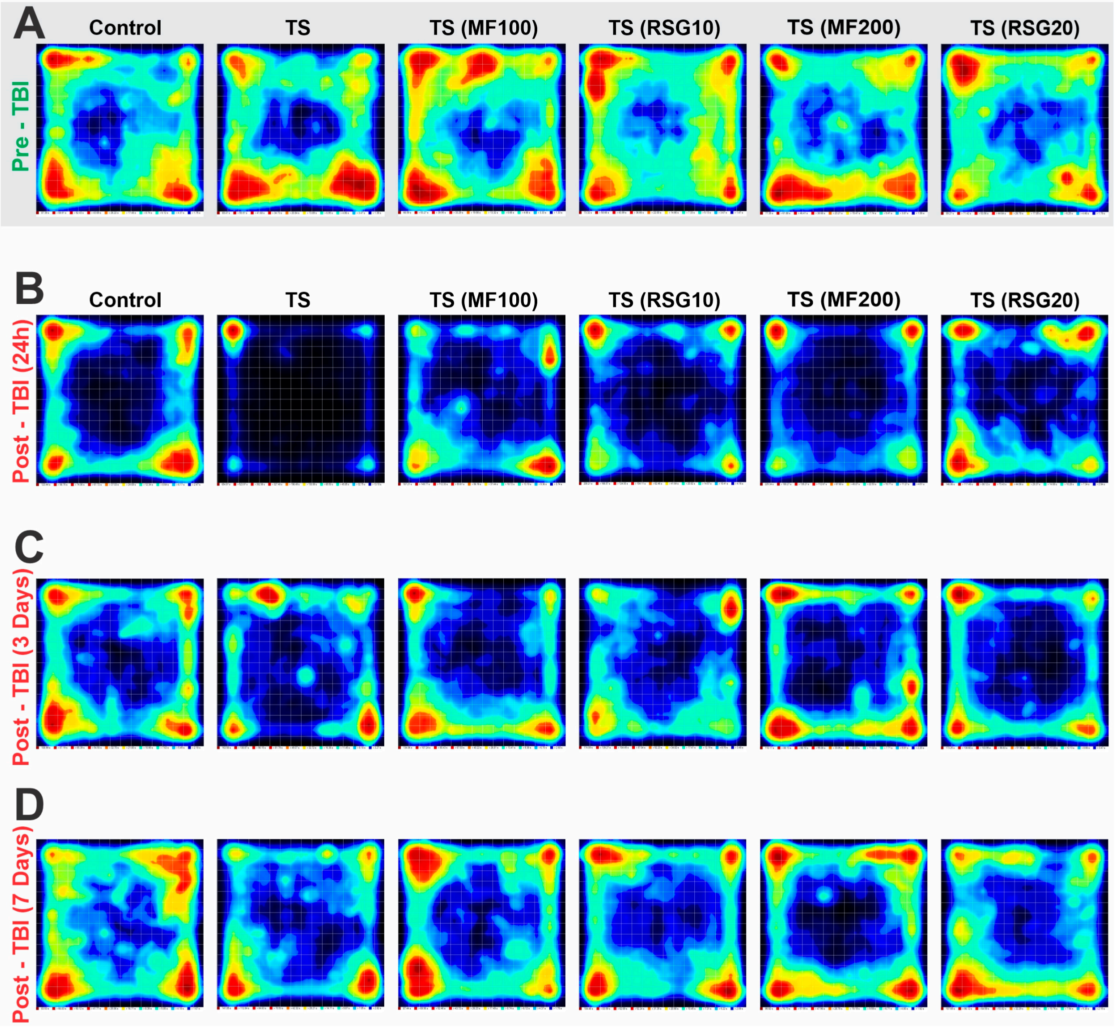

Figure S1. Animals' walking patterns and exploratory behavior: (**A**) Pre-TBI walking patterns of chronically exposed TS mice show the most traveled distance with the most movements and very low resting time. However, the walking patterns 24 hr post-TBI clearly show less traveled distance and movements and extended resting time for un-treated TS-exposed mice (**B**). Walking patterns assessed at 3 days (**C**) and 7 days (**D**), post-TBI show a similar trend where animals receiving MF or RSG recovered their motor activity and exploratory behavior compared to non-treated animals.

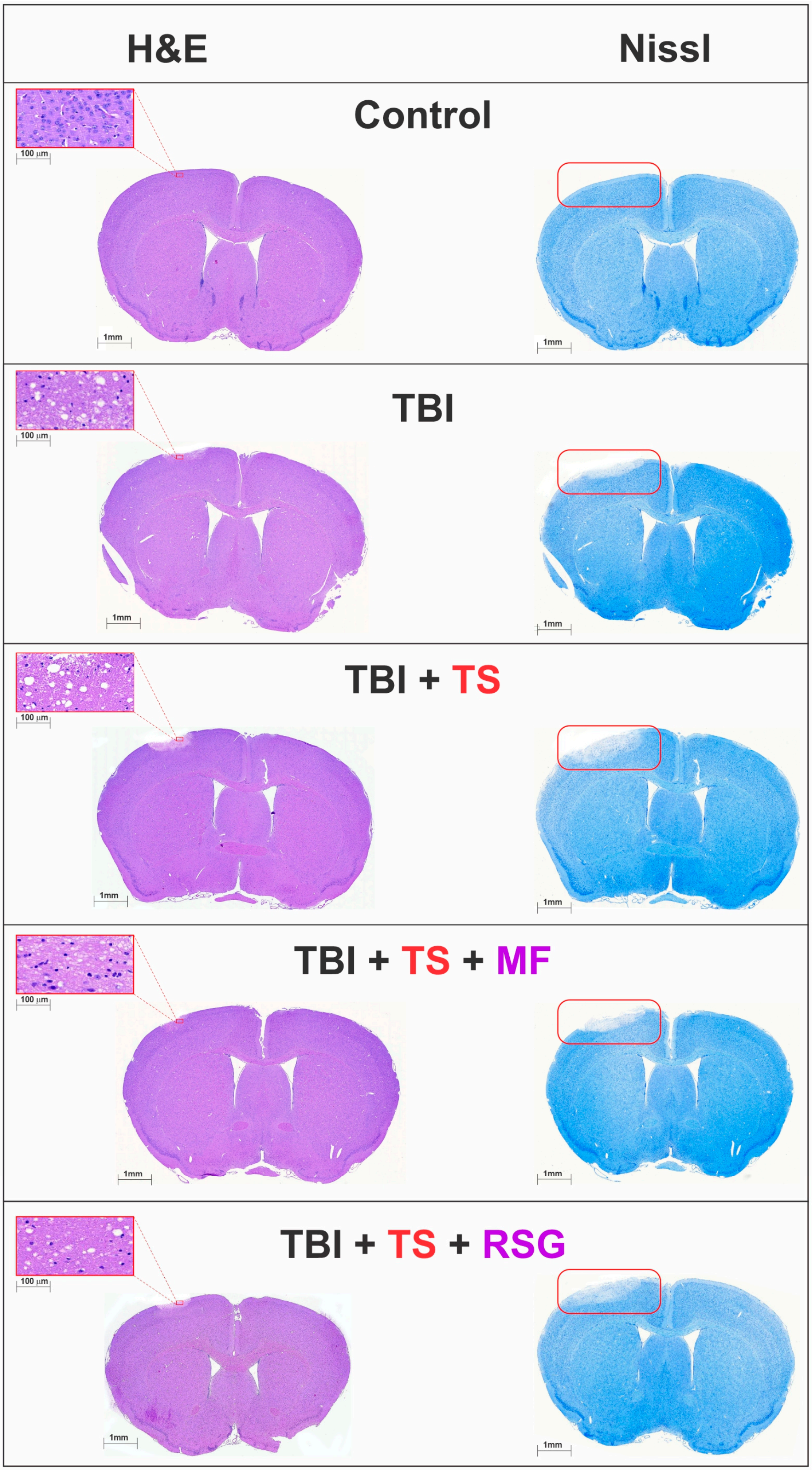

Figure S2. Side by side H&E stains (Left) and Nissl stains (Right) of mouse brain tissue slices from animals sacrificed seven days post-TBI to collect blood and brain samples for subsequent biochemical, histological, and molecular assessments. Samples include control (no TBI) and the main 4 test groups corresponding to TBI, TBI+TS, TBI+TS+MF200 (highest dose tested), and TBI+TS+RSG20 (highest dose tested). The high magnification

insets over the corresponding H&E stains are enlarged images corresponding to the regions marked by the red boxes in the affected areas.

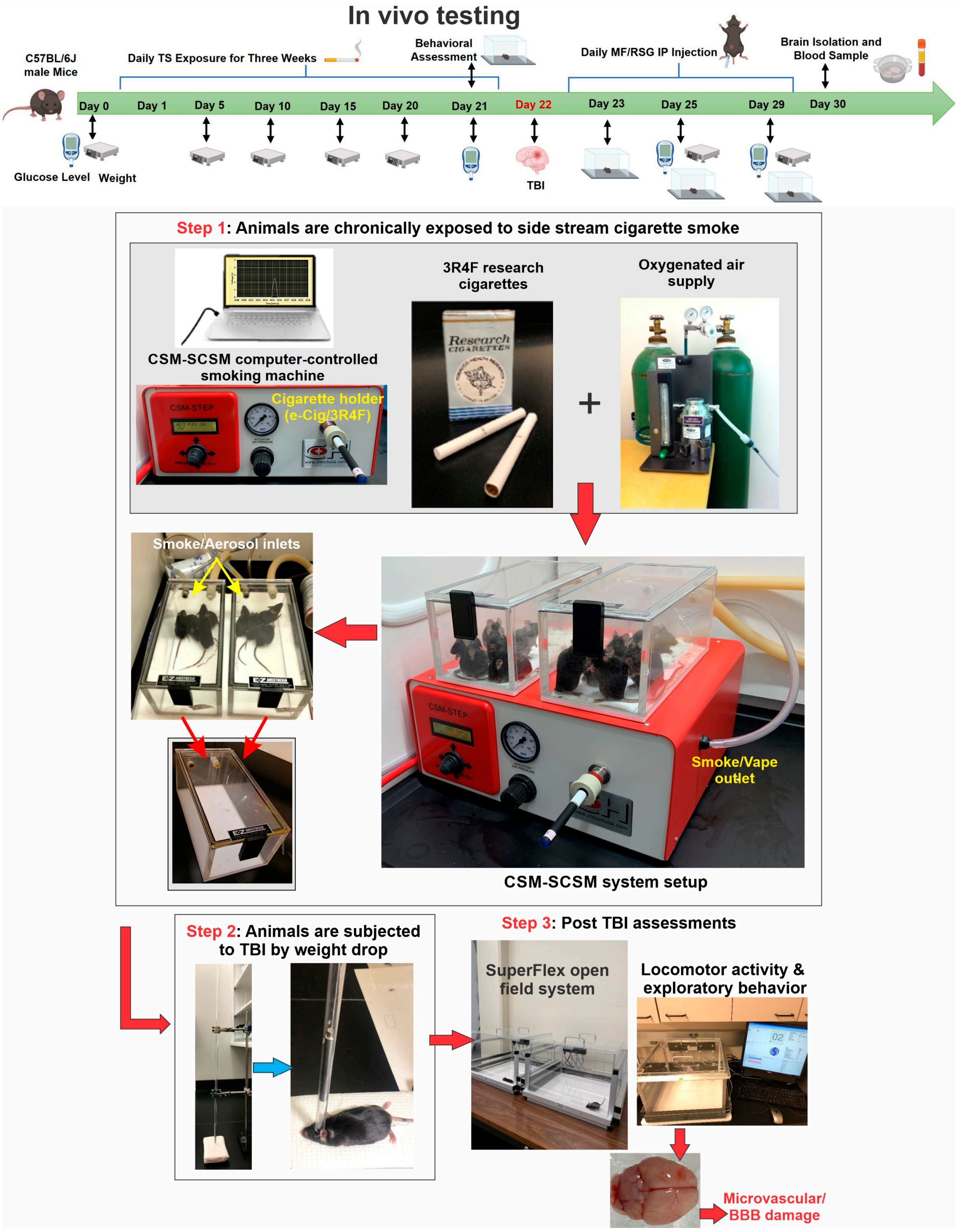

Figure S3. (A) Timeline of the *in vivo* study. The mice were divided into six groups (6 mice/group), including TBI Control, TBI+TS, TBI+TS+MF100, TBI+TS+MF200, TBI+TS+RSG10, and TBI+TS+RSG20. Mice were exposed to TS for a total of three weeks. On day 22, all groups were subjected to TBI. Following TBI, MF/RSG was administered to the corresponding groups for 7 days. Behavioral analyses, fasting blood glucose levels, and body weight measurements were performed as indicated. On day 30, mice were sacrificed, and the brain tissues and blood samples were extracted for subsequent biochemical and molecular assessments. **(B) Experimental flow and setup, including cigarette smoke generation, TS mice exposure, TBI induction, and post-traumatic motor activity assessment**. C57BL/6J male mice were

chronically exposed to TS (full body side stream exposure) for three weeks using a computer-controlled single cigarette smoking machine using the FTC-approved cigarette smoking protocol. Test animals were subjected to TBI by a drop of the head weight (30 g) from an 80 cm height through a pre-positioned vertical guide. The physical activity of the mice was analyzed before TBI and at 24 h, three days, and seven days after TBI using an open-field test. Finally, mice were sacrificed to collect blood and brain samples for subsequent biochemical, molecular, and histopathological assessments.
